# Application of One-Step IADPSG Versus Two-Step Diagnostic Criteria for Gestational Diabetes in the Real World: Impact on Health Services, Clinical Care, and Outcomes

**DOI:** 10.1007/s11892-017-0922-z

**Published:** 2017-08-10

**Authors:** Florence M. Brown, Jennifer Wyckoff

**Affiliations:** 1 Joslin Diabetes Center, 1 Joslin Pl, Boston, MA 02215 USA; 2 Harvard Medical School, Boston, USA; 3 University of Michigan, Ann Arbor, USA

**Keywords:** GDM, IADPSG, Diagnosis, Prevalence, Outcomes, Cost

## Abstract

**Purpose of Review:**

This paper seeks to summarize the impact of the one-step International Association of Diabetes and Pregnancy Study Groups (IADPSG) versus the two-step gestational diabetes mellitus (GDM) criteria with regard to prevalence, outcomes, healthcare delivery, and long-term maternal metabolic risk.

**Recent Findings:**

Studies demonstrate a 1.03–3.78-fold rise in the prevalence of GDM with IADPSG criteria versus baseline criteria. Women with GDM by IADPSG criteria have more adverse pregnancy outcomes than women with normal glucose tolerance (NGT). Treatment of GDM by IADPSG criteria may be cost effective. Use of the fasting glucose as a screen before the 75-g oral glucose tolerance test to rule out GDM with fasting plasma glucose (FPG) < 4.4 (80 mg/dl) and rule in GDM with FPG ≥ 5.1 mmol/l (92 mg/dl) reduces the need for OGTT by 50% and its cost and inconvenience. The prevalence of postpartum abnormal glucose metabolism is higher for women with GDM diagnosed by IADPSG criteria versus that for women with NGT.

**Summary:**

Data support the use of IADPSG criteria, if the cost of diagnosis and treatment can be controlled and if lifestyle can be optimized to reduce the risk of future diabetes.

## Introduction

For well over 50 years, there has been a lack of consensus over the appropriate diagnostic criteria for gestational diabetes mellitus (GDM) and the significance of the diagnosis. Competing diagnostic criteria across the globe have complicated the delivery of healthcare and the design and interpretation of research in GDM [1•, 2]. The Hyperglycemia and Adverse Pregnancy Outcomes (HAPO) study was intended to lead to unification and agreement on the diagnostic criteria for GDM [3]. In 2010, the International Association of Diabetes and Pregnancy Study Groups (IADPSG) released their recommendations for a new set of diagnostic criteria, based on the HAPO study outcomes [4]. However, an ongoing global debate continues about when and how to screen and diagnose GDM. A variety of local, regional, and institutional diagnostic criteria continues to be applied in practice, confusing both healthcare delivery and research [1•, 5]. This chapter seeks to review this history and explore the outcomes and impact of the IADPSG criteria.

## Evolution of the Diagnostic Criteria for GDM

### O’Sullivan and Mahan

In the 1960s, pregnancy was generally known to induce a state of reduced glucose tolerance. Whether this was pathologic was debated. How this should be assessed, by intravenous glucose tolerance test (IGTT) versus oral glucose tolerance test (OGTT), was also unclear. Furthermore, what glucose level should be considered abnormal was unknown.

At the 23rd annual meeting of the American Diabetes Association (ADA) in Atlantic City on June 16, 1963, John O’Sullivan and Clare Mahan from Boston City and the Boston Lying-In hospitals presented their landmark study demonstrating the ability of an OGTT performed during pregnancy to predict future risk of type 2 DM (DM2) [6]. They followed 752 women with baseline 100-g 3-h OGTTs performed at registration with the obstetric clinic, with periodic OGTTs for 8 years, and compared the predictive value of three glucose levels representing the 84.1 (level I), 97.7 (level II), and 99.9th (level III) percentiles (1, 2, and 3SD above the mean, respectively). The authors considered two or more values that met or exceeded targets to be a positive test. They reported that the cumulative incidence of DM2 after approximately 8 years was 17.2% for level I, 29% for level II, and 60.1% for level III. The authors chose the level II criteria (2SD above the mean) as the best choice for predicting the development of DM2 as it reflected the results of the cortisone OGTT [7], a method shown at that time by Conn and Fajans to predict the development of DM2 and the prevalence of DM2 in a community [8]. Subsequently, a 16-year cumulative incidence of DM2 of 60% was demonstrated by life table analysis in the level 2 group [9]. A 1-h 50-g glucose challenge test (GCT) with whole blood glucose threshold of 7.2 mmol/l (130 mg/dl) had 87% specificity and 79% sensitivity for stratifying risk in a population with 2.5% prevalence of GDM [9]. The O’Sullivan and Mahan paper (and its recommendation of “level II” criteria for the diagnosis of GDM) was the cornerstone of GDM diagnosis in the USA for the next 40 years. These criteria are described in Table 1.

**Table 1:** Major criteria for the diagnosis of GDM

	Sample	Steps	OGTT load	No. abnormal	Fasting mg/dl (mmol/l)	1 h mg/dl (mmol/l)	2 h mg/dl (mmol/l)	3 h mg/dl (mmol/l)
O’Sullivan 1964 [6]	B	2	100 g	≥2	90 (5)	165 (9.2)	145 (8.1)	125 (6.9)
NDDG 1979 [11]	P	2	100 g	≥2	105 (5.8)	190 (10.6)	165 (9.2)	145 (8.0)
C&C 1982 [12]	P	2	100 g	≥2	95 (5.3)	180 (10)	155 (8.6)	140 (7.8)
EASD1996 [15]	P	1	75 g	≥1	108 (6)	X	162 (9)	X
ADIPS1998 [16]	P	1	75 g	≥1	100 (5.5)	X	144 (8)	X
WHO 1999 [17]	P	1	75 g	≥1	126 (7)	X	140 (7.8)	X
IADPSG 2010 [4]	P	1	75 g	≥1	92 (5.1)	180 (10)	153 (8.5)	X

X= not applicable

B= Whole blood

P= Plasma

### National Diabetes Data Group (NDDG)

The O’Sullivan criteria were based on the measurement of glucose in whole blood [6]. Glucose measured in whole blood tended to be less accurate than glucose measured in plasma or serum in part because blood cells would metabolize about 5% of the glucose in the sample per hour [10]. In the 1970s, laboratories moved to measure glucose in plasma. However, glucose measured in whole blood was generally 15% lower than glucose measured in plasma. So, the O’Sullivan criteria needed to be revised to reflect the change in glucose measurement. In 1979, the NDDG endorsed a revision of the O’Sullivan criteria reflecting this change in measurement technique [11].

### Carpenter and Coustan (CC)

In 1982, Carpenter and Coustan suggested a new set of diagnostic criteria based on an adjustment of O’Sullivan’s criteria taking into account the measurement of plasma glucose using the glucose oxidase method instead of the previously used Somogyi-Nelson method [12]. This created two different interpretations of O’Sullivan and Mahan’s criteria based on the 100-g OGTT in the USA. The CC criteria diagnosed 30–50% more women with GDM than the NDDG criteria [13]. Recently, in a secondary analysis of Landon et al.’s landmark study of the impact of treatment of GDM, Harper found that those diagnosed by CC criteria benefitted equally from treatment compared with those diagnosed by NDDG criteria. The number needed to treat to prevent a single outcome of cesarean delivery (CS), pregnancy-induced hypertension (PIH), large-for-gestational-age infant (LGA), and macrosomia was 20 or less for the overall group and similar for both CC and NDDG criteria [13, 14].

### International Criteria

While the two-step method using a 50-g GCT as a screening test followed by a 100-g OGTT (utilizing either CC or NDDG criteria) was widely adopted in the USA, much of the rest of the world preferred a one-step screening 75-g OGTT method. Between 1990 and 2005, there was a proliferation of competing international criteria, many based on a one-step method using a 75-g OGTT, with published guidelines from the European Association for the Study of Diabetes (EASD) in 1996 [15], the Australasian Diabetes in Pregnancy Society (ADIPS) in 1998 [16], and the World Health Organization (WHO) in 1999 [17], among others [1•, 18]. A large-scale study of maternal hyperglycemia and its relationship to fetal outcomes was needed to try to resolve these differences.

### The Hyperglycemia and Adverse Pregnancy Outcomes (HAPO) Study

The HAPO study was designed with the goal to achieve consensus in the diagnosis of GDM by investigating the impact of maternal glycemia, less severe than overt diabetes, on the risk of adverse pregnancy and neonatal outcomes [3]. Consistent with previous studies [19, 20], the HAPO study demonstrated a linear increase in the risk of primary outcomes (LGA, cord C-peptide, clinical neonatal hypoglycemia (NH), and primary CS) with increasing degrees of hyperglycemia, as assessed by a fasting 75-g OGTT at fasting, 1 and 2 h. Secondary outcomes of preterm delivery (PTD) < 37 weeks’ gestation, shoulder dystocia/birth injury, and preeclampsia also increased with increasing glucose levels [3] as did neonatal skinfold thicknesses > 90% [21].

### The International Association of Diabetes in Pregnancy Study Groups (IADPSG)

The IADPSG convened a consensus conference in June 2008 with the goal of reaching international consensus regarding the challenging aspects of the diagnosis of GDM, including:

1. How to use the HAPO findings to create diagnostic criteria for GDM based on pregnancy and neonatal outcomes
2. How to establish the one-step 75-g OGTT as the preferred international diagnostic test for GDM
3. How to screen and diagnose preexisting DM (PEDM) in the first trimester

The IADPSG recommendations were published in 2010 [4].

1. How to use the outcomes from HAPO to create a diagnostic criteria for GDM based on pregnancy and neonatal outcomes

The first challenge was to establish dichotomous targets for the fasting, 1- and 2-h values even though the risks of adverse outcomes demonstrated strong linear relationships with these variables. An odds ratio of 1.75 times the mean was selected for the outcomes of increased neonatal body fat, LGA, and cord c-peptide greater than the 90th percentile to arrive at the recommended diagnostic criteria for GDM. The plasma glucose levels corresponding to or above an OR of 1.75 were fasting ≥ 5.1 mmol/l (92 mg/dl); 1 h ≥10 mmol/l (180 mg/dl); 2 h ≥ 8.5 mmol/l (153 mg/dl) [4]. GDM was diagnosed if one or more values were met and were less than the criteria for overt diabetes [4]. Using these criteria, the prevalence of GDM in the collaborating HAPO centers ranged from 9.3–25.5%, with an average of 17.8% for the study overall [22]. The chosen cutoffs were necessarily arbitrary and based on expert opinion. In part, due to the linear relationship of glucose levels with higher frequencies of adverse outcomes, the choice of IADPSG thresholds has been debated, balancing risks and costs against benefit.

2) Establish the one-step 75-g OGTT over the two-step 100-g OGTT as the preferred international diagnostic test for GDM

A survey of IADPSG members indicated that 60% opted for the one-step method, while 30% preferred the two-step method [23]. However, to convince healthcare delivery institutions and health professional associations that advocate the highest standards of practice that the one-step 75-g OGTT is superior to the two-step 100-g OGTT for the diagnosis of GDM, studies are needed to demonstrate the effect of treatment on short-term pregnancy and neonatal outcomes, as well as long-term cardiometabolic benefits to mother and offspring and the cost effectiveness of treatment.

3) Make recommendations on how to screen and diagnosis PEDM in the first trimester

The American Congress of Obstetricians and Gynecologists (ACOG) acknowledged that the benefit of treatment of GDM identified early in pregnancy has not been demonstrated but rather has been accepted on a theoretical basis [24]. Prior to the IADPSG recommendations, ACOG recommended two-step screening for GDM in the first trimester for women considered to be high risk [24]. Risk factors for GDM include the following: > 35 years old, overweight or obese, chronic hypertension or polycystic ovarian syndrome, prior GDM, strong family history of diabetes, stillbirth in a previous pregnancy, and high-risk racial/ethnic group (African American, American Indian, Asian American, Hispanic, Latina, or Pacific Islander).

At the IADPSG conference, there was consensus on the need to diagnose women meeting criteria for PEDM in the first trimester defined by fasting plasma glucose (FPG) ≥ 7.0 mmol (126 mg/dl), random blood glucose (RBG) ≥ 11.1 mmol/l (200 mg/dl), or hemoglobin A1c (A1c) ≥ 48 mmol/mol (6.5%), but there was no consensus on universal early screening [4]. They defined early diagnosis of GDM based on the same criteria for GDM later in pregnancy, if not diagnostic of PEDM, with FPG ≥ 5.1 mmol/l (92 mg/dl) but ≤ 7.0 mmol/l (126 mg/dl). If early pregnancy FPG was ≤ 5.1 mmol/l (92 mg/dl), they recommended repeat testing for GDM from 24 to 28 weeks’ gestation with a 75-g OGTT. They acknowledged that there was no data to support this recommendation.

The IADPSG criteria were erratically adopted for both the first trimester and 24–28-week screening, and instead of creating consensus, they became yet another set of competing criteria, which further added to the confusion.

### NIH Consensus Conference

Following the release of the IADPSG recommendations, an NIH Consensus Conference was convened to respond to the controversies in the screening and diagnosis of GDM [25]. In preparation, the Agency for Healthcare Research and Quality (AHRQ) contracted with the University of Alberta Evidence-based Practice Center to create an evidence-based report on this topic [26]. Their search identified 14,398 citations and included 97 studies (6 randomized controlled trials (RCTs), 63 prospective cohort studies, and 28 retrospective cohort studies).

This exhaustive review revisited some of the key points of GDM diagnosis and the IADPSG consensus recommendations. In review of the first trimester screening for PEDM, they found limited evidence for or against early screening [25, 26]. For universal second trimester screening, they concluded that while there was clear evidence of worsening pregnancy and neonatal outcomes with increasing levels of glucose, there was no adequate data to support a one-step 75-g OGTT over the two-step 100-g OGTT [25, 26]. Financial implications were not addressed.

## Impact of the Application of IADPSG Criteria

### Prevalence of GDM With the Application of IADPSG Criteria

**Fig. 1:**
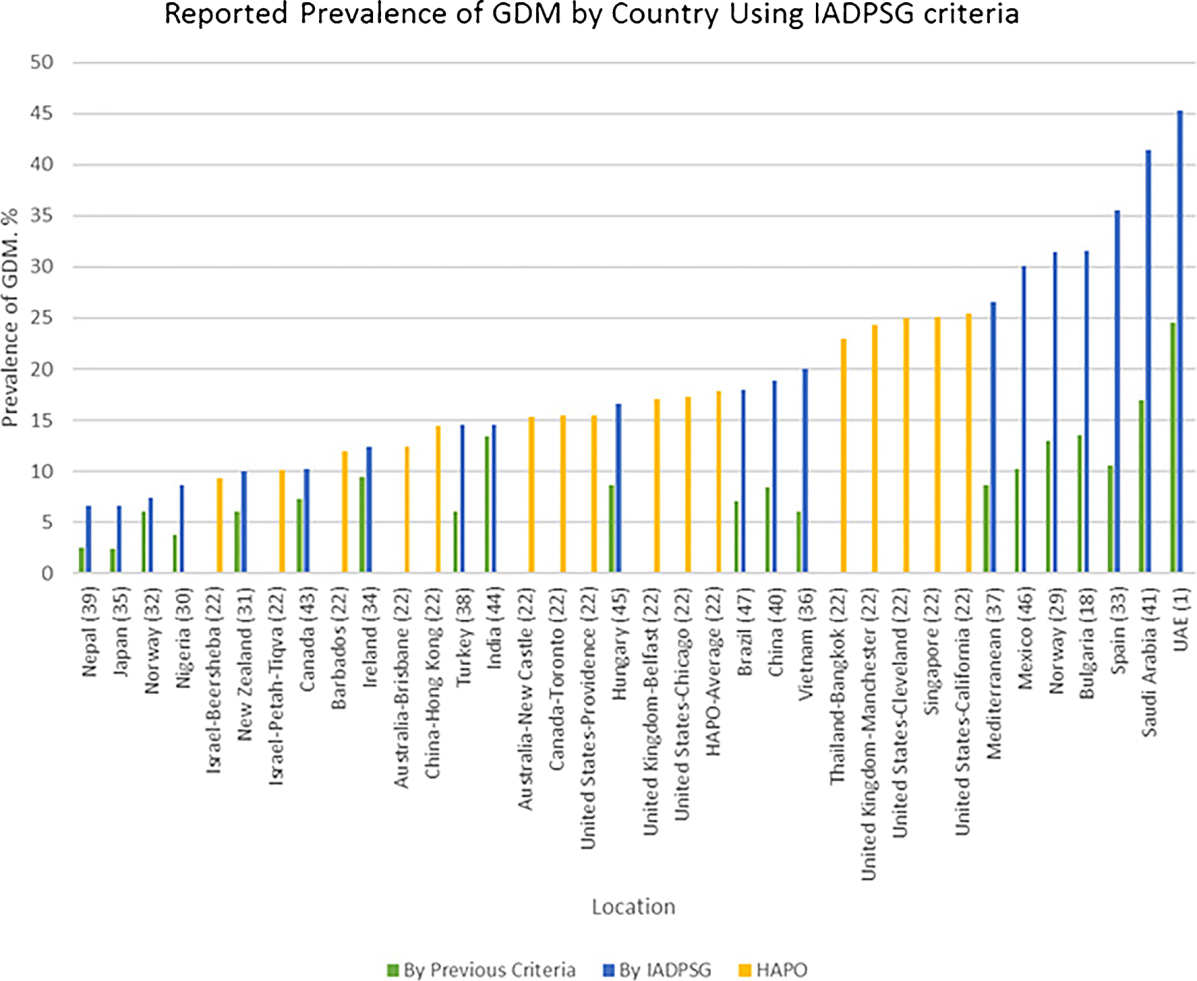
Prevalence of GDM by country using the IADPSG criteria

**Fig. 2:**
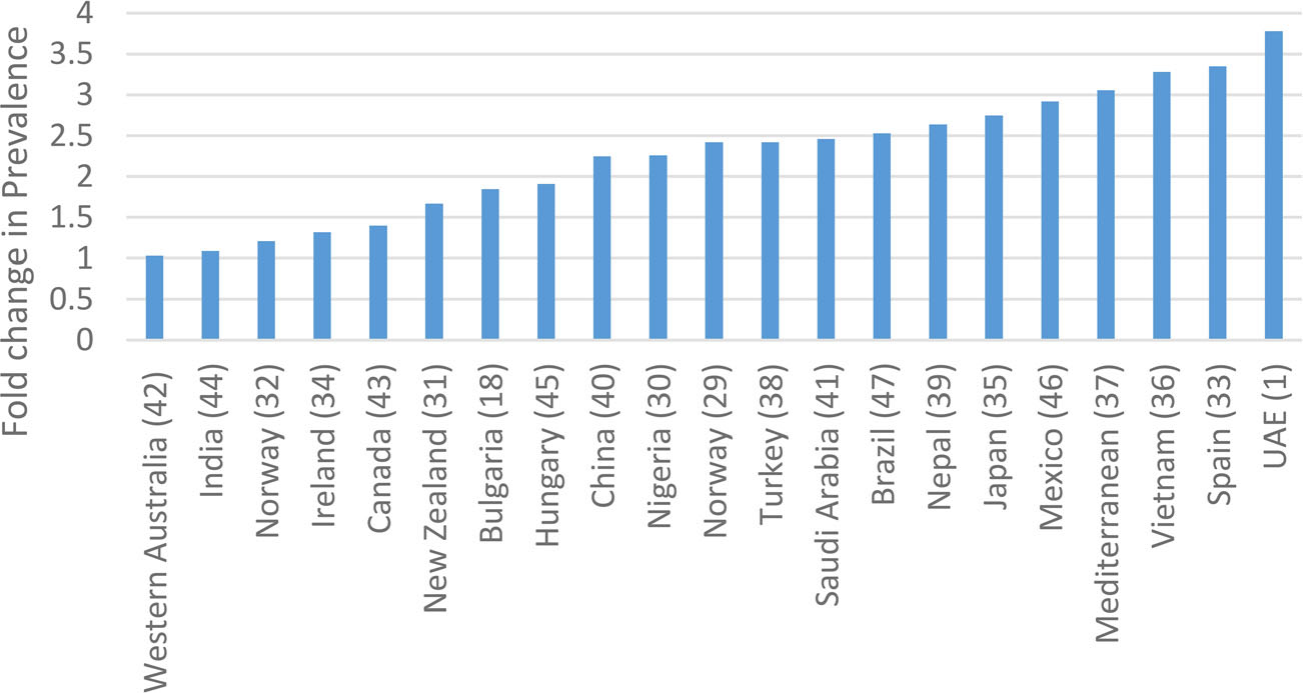
Fold change in prevalence

The prevalence of GDM using consistent criteria over time has been increasing globally, mirroring the epidemic of DM2 and obesity [27, 28]. Comparing GDM prevalence between countries has been confounded by differing sets of diagnostic criteria. In an illustrative 2012 international survey, Jiwani details the huge variation in screening practices [5]. HAPO provided some of the first robust international comparisons of GDM prevalence. Applying the IADPSG criteria retrospectively to the HAPO study population, the frequency of GDM was 17.8% overall, with a range from 9.3 to 25.5% in the collaborating centers [22]. The IADPSG consensus panel had predicted that the prevalence of GDM using the IADPSG criteria would be higher compared with that of most other criteria in use throughout the world. So, the introduction of IADPSG criteria led to a plethora of data looking at local prevalence of GDM [1•, 18, 29–32, 33••, 34–47]. Figure 1 compares reported prevalence rates for GDM from various countries using the IADPSG criteria in comparison to the criteria previously applied in that country. The reported prevalence of GDM using IADPSG criteria varied from 3.5 to 45.3% [1•, 39]. IADPSG universally increased the prevalence. The absolute increase was by as much as 33% [1•]. Figure 2 shows the fold change from baseline criteria ranged from 1.03 to 3.78 [1•, 18, 29–32, 33••, 34–47]. Table 2 describes the data.

**Table 2:** Change in prevalence of GDM using the IADPSG criteria

Country	Baseline prevalence			IADPSG	Absolute change from local reference criteria	Fold change from local criteria
	ADA^a^	WHO^b^ 1999	Regional			
Norway [29]		13%		31.5%^a^	+18.5%	2.42
UAE [1•]	12%	24.5%		45.3%	+33.3%	3.78
Nigeria [30]		3.8%		8.6%	+4.8%	2.26
New Zealand [31]			6%^c^	10%	+4%	1.67
Norway [32]		6.1%		7.4%^a^	+1.3%	1.21
Brazil [47]		7.1%		18%	+10.9%	2.53
Spain [33••]	10.6%			35.5%	+24.9%	3.35
Ireland [34]		9.4%		12.4%	+3%	1.32
Japan [35]			2.4%^d^	6.6%	+4.2%	2.75
Vietnam [36]	6.1%			20%	+13.9%	3.28
Mediterranean [37]	8.7%			26.6%	+17.9%	3.06
Turkey [38]	6%			14.5%	+8.5%	2.42
Nepal [39]		2.5%		6.6%	+4.1%	2.64
China [40]	8.4%			18.9%	+10.5%	2.25
Saudi Arabia [41]			16.9%^e^	41.5%	+24.6%	2.46
Western Australia [42]			3.4%^f^	3.5%	+0.1%	1.03
Canada [43]			7.3%^g^	10.2%	+2.9%	1.40
India [44]		13.4%		14.6%	+1.2%	1.09
Hungary [45]		8.7%		16.6%	+7.9%	1.91
Mexico [46]	10.3%			30.1%	+19.8%	2.92
Bulgaria [18]	13.5%	17.1%		31.6%	+18.1%	1.85

^a^Two-step 100-g OGTT using either CC or NDDG criteria

^b^World Health Organization (WHO)

^c^Using New Zealand Society for the Study of Diabetes (NZSDD) criteria

^d^Using Japan Society for Obstetrics and Gynecology (JSOG) criteria

^e^Two-step ADA criteria but a 75-g OGTT

^f^Using Australasian Diabetes in Pregnancy Society (ADIPS) criteria

^g^Using Canadian Diabetes Association (CDA) 2008

In two elegant studies, authors compared the prevalence of GDM in their respective hospitals, using a variety of criteria: ADA 2003, ADIPS 1998, CDA 2013, European Association for the Study of Diabetes (EASD) 1996, IADPSG 2010, NZSSD 2004, and WHO 1999 [1•, 18]. In the Bulgarian study, 800 women were screened using a 75-g OGTT [18]. The prevalence of GDM varied from 10.8% using the EASD criteria to 31.6% using the IADPSG criteria. In the United Arab Emirates (UAE), 2337 women were screened with a 75-g OGTT between 24 and 28 weeks’ gestation [1•]. Most (97.6%) of this study population were either of Arabic or Indian descent. A historical data set from the same hospital in 2010 when the 100-g two-step method was being applied was used to compare prevalence to NDDG and CC criteria. Prevalence using the one-step method ranged from 9.2% (CDA 2003) to 45.3% (IADPSG), a 5-fold difference. Furthermore, use of IADPSG criteria resulted in a 6-fold higher prevalence compared to a historical prevalence of 7.7%, when NDDG criteria were applied.

Two Norwegian studies demonstrate that there are aspects to study design other than diagnostic criteria that need to be considered when trying to understand the differences in the prevalence of GDM between studies, including percentage of population screened, universal versus selective screening, and gestational age at testing [29, 32]. In an analysis of data from a previously reported RCT of a structured exercise program to prevent GDM, 852 Caucasian women were screened using a 75-g OGTT between 18 and 22 weeks and then again between 32 and 36 weeks [32]. The prevalence of GDM at 32–36 weeks was 6.1% for WHO criteria versus 7.4% for IADPSG criteria. IADPSG criteria resulted in only a 1.2-fold increase in prevalence of GDM over WHO 1999 criteria. In total, 73 women (10.6%) were diagnosed with GDM: 22 by WHO only, 31 by IADPSG only, and 20 (27%) by both. Important considerations in the design of this study include the ethnic composition, the population being drawn from a group of women voluntarily participating in an exercise study which excluded high-risk pregnancies, the exercise intervention of the study itself, etc.

In a second population-based cohort study from Norway, 759 pregnant women (59% of whom were from ethnic minorities), were screened at 26–30 weeks using modified IADPSG criteria [29]. A 2.4 times greater GDM prevalence was found using IADPSG of 31.5% compared to 13% when WHO 1999 criteria were applied. If only the subset of women of Western European origin was analyzed, the prevalence with IADPSG was 24% and that with WHO 1999 11%.

In summary, studies demonstrate a 1.03–3.78-fold rise in the prevalence of GDM with the IADPSG criteria compared with baseline criteria.

### Impact of One-Step IADPSG Versus Two-Step Diagnostic Criteria for Gestational Diabetes on Outcomes

Treatment of GDM has been shown to reduce adverse outcomes [14, 48]. The Australian Carbohydrate Intolerance Study in Pregnant Women (ACHOIS) demonstrated that pregnancies in women diagnosed with GDM using the WHO 1999 criteria and randomized to treatment with education, diet, and, if necessary to achieve goals, insulin resulted in lower rates of serious perinatal complications (1 vs 4%, *p* = 0.01), macrosomia (10 vs 21%, *p* < 0.001), and preeclampsia (12 vs 18%, *p* = 0.02) [48]. In a study based in the USA, Landon et al. demonstrated that pregnancies in women diagnosed with GDM using the CC criteria and randomized to treatment with education, diet, and, if necessary to achieve goals, insulin resulted in lower rates of macrosomia (5.9 vs 14.3%, *p* < 0.001) and preeclampsia (8.6 vs 13.6%, *p* = 0.01) [14].

After the publication of the IADPSG criteria, several studies investigated the risk of adverse outcomes in pregnancies complicated by GDM using the new criteria. Prospective observational studies [36, 38, 49•] and a retrospective observational study [50] comparing women diagnosed with GDM using the IADPSG criteria but untreated (excluding those who also met CC criteria) to women with NGT found an increased risk of polyhydramnios [38], preeclampsia [49•], PTD [36], primary CS [49•], neonatal hypoglycemia [36], LGA [49•, 50], cord C-peptide [49•], and newborn percentage body fat above the 90th percentile [49•]. Infants also had higher ponderal index and *z* scores [50]. Women with untreated GDM by IADPSG criteria had larger birth weight infants than women with treated GDM by CC criteria [50].

A prospective study comparing untreated women diagnosed with GDM using the IADPSG criteria (excluding those who met WHO criteria) to women with NGT found higher rates of preeclampsia, polyhydramnios, LGA, CS, neonatal intensive care (NICU) admission, and neonatal hypoglycemia [51].

Studies comparing women diagnosed with GDM using the IADPSG criteria but untreated (excluding other regional criteria) to women with NGT also found higher risk of adverse outcome [35, 43, 52] with one exception [53].

A retrospective cohort study investigated the impact of different glycemic thresholds on outcomes in women screened with a 75-g 2-h OGTT [54]. Glycemic thresholds were based on outcomes of the HAPO study (OR 1.75 (GDM1) vs OR > 2.0 (GDM2)). Women with one abnormal value were not treated. Women were excluded and treated if they had two abnormal values exceeding fasting ≥ 5.5 mmol/l (100 mg/dl); 1-h 10.6 mmol/l (195 mg/dl); and 2-h 8.9 mmol/l (160 mg/dl). GDM1 had one abnormal glucose value in ranges for fasting 5.1–5.2 mmol/l (92–94 mg/l); 1-h 10–10.6 mmol/l (180–190 mg/dl); 2-h 8.5–9.0 (153–162 mg/dl). GDM2 had one abnormal glucose value with fasting ≥5.3 mmol/l (95 mg/l), 1-h > 10.6 mmol/l (≥191 mg/dl), 2-h > 9.0 mmol/l (≥162 mg/dl). Compared with NGT, GDM1 had increased birth weight and LGA. Compared with NGT, GDM2 had increased risk of preeclampsia/eclampsia, PTD, CS, shoulder dystocia, increased birthweight, LGA, and neonatal hypoglycemia. These findings indicate more adverse outcomes when HAPO glucose values for OR 2.0 are used as thresholds rather than IADPSG OR 1.75 thresholds. Authors cite the need for an RCT to determine benefit from treatment in these different groups of mild GDM [54].

Adverse outcomes of untreated GDM based on IADPSG criteria varied depending on whether the fasting glucose or the post glucose load was abnormal. An elevated fasting with normal post glucose load values was associated with increased LGA, while elevated post glucose loads with a normal fasting were associated with increased preterm delivery, gestational hypertension, and hyperbilirubinemia when compared with NGT [55].

Studies comparing the effects of treated GDM on outcomes for women diagnosed by IADPSG criteria versus prior/usual criteria including a modified 75-g CC [41], NDDG [56], or a modified two-step WHO 1999 [57] demonstrated similar pregnancy and neonatal outcomes as those diagnosed by CC and NDDG criteria, but greater risk of macrosomia and LGA compared with WHO criteria. These results suggest that IADPSG criteria may diagnose women with similar risk of adverse outcomes as CC and NDDG, but may identify women at higher risk of adverse outcomes than WHO. In addition, the treated IADPSG only group (excluding those diagnosed by modified CC criteria) had increased risk of CS, Apgar score < 7 at 5 min, and neonatal hypoglycemia compared with the NGT group [41].

Women diagnosed with GDM by IADPSG criteria had similar prevalence of risk factors as women diagnosed by CC, NDDG, or WHO criteria. Risk factors studied included South Asian high-risk ethnic group [29], prepregnancy overweight [29, 41], maternal age [32, 41], fasting insulin [32], no regular exercise [32], and one or more ACOG risk factors [56].

In summary, women diagnosed with GDM by IADPSG criteria have higher risk of adverse pregnancy and neonatal outcomes and higher prevalence of risk factors than woman with NGT.

### Maternal Risk for Type 2 Diabetes Based on the IADPSG Criteria

As noted, O Sullivan’s original work was intended to predict the development of DM2 in the mother [6]. As diagnostic criteria evolved, further studies described the predictive ability of the varying sets of criteria. Women with a history of GDM had approximately a 50% risk of developing type 2 DM within 7–10 years [58] of their pregnancy and a 7 times increased risk compared to women with NGT [59].

The IADPSG criteria have also been shown to predict maternal postpartum glucose abnormalities [60•, 61]. In the ATLANTIC-DIP study of white Europeans, the results of 75-g OGTTs at 12 weeks’ postpartum were compared between 270 women with a history of GDM based on IADPSG criteria and 388 women with normal glucose tolerance during pregnancy [60•]. In total, at 12 weeks’ postpartum, 15.6% of women with previous history of GDM demonstrated glucose abnormalities (14.1% IFG and/or IGT and 1.5% DM2) compared to 0.8% of women with no history of GDM. This cohort was followed for up to 5 years, (a mean follow-up for those with GDM of 2.6 years), and at repeat testing another 10.4% of those with a history of GDM had developed abnormal glucose levels compared to only 2.8% of those without history of GDM. This resulted in a cumulative percentage of 25.9% of those with a history of GDM having glucose abnormalities compared to only 3.6% of those with no history of GDM.

In a study of 305 ethnically homogenous Czech women, the postpartum prevalence of abnormal glucose in women diagnosed with GDM was 16.7% [61]. Based on receiver operating curves (ROC) analysis, this study concluded that the IADPSG cutoff values performed better than WHO for risk stratification of postpartum glucose abnormality (Table 3).

**Table 3:** Maternal risk of type 2 diabetes based on IADPSG criteria

DM criteria	Ref	Ethnicity	Time point	No GDM, risk of IFG/IGT, %	GDM, risk of DM2, %	GDM, risk of IFG/IGT, %
O’Sullivan	[6]	US	8 years	Not reported	29	Not reported
O’Sullivan	[9]	US	16 years	Not reported	60	Not reported
NDDG	[84]	US	6 weeks	Not reported	2.6	6.8
IADPSG	[60•]	Irish	12 weeks	0.8	1.5	14.1
IADPSG	[60•]	Irish	2.5 years	3.6	Not reported	25.9
IADPSG	[61]	Czech	12 weeks	Not reported	5.2	10.5

In summary, studies up to 5 years’ postpartum indicate an increased risk of impaired glucose metabolism in women who had GDM by IADPSG criteria, compared to that in women with NGT.

### Financial Implications of IADPSG

The organizers of the Pasadena conference understood the IADPSG criteria would increase the prevalence of GDM. This invariably led to the question of the financial implications and cost effectiveness of treating those diagnosed by the new set of criteria.

In 2012, Agarwal et al. compared the costs and laboratory work load units (WLUs) of using the 50-g GCT screening test followed by the 100-g OGTT using CC criteria with the one-step 75-g OGTT in a high-risk population in the UAE [62•]. In a 12-month study, 1101 women were tested. Standard insurance billing was $18.60 for a 50-g GCT and $50 for either a 75- or 100-g OGTT. Testing under the one-step method resulted in $55,250 in billing compared to $31,985 using the two-step method. However, annual WLUs were only 18,662 using the one-step method versus 28,975 using the two-step method. They concluded that adoption of the one-step method would increase the cost by 42% but would decrease laboratory workload by 36%.

In a cost-effectiveness analysis, Mission et al. compared the one-step IADPSG criteria versus the two-step CC criteria using a Monte Carlo simulation to assess cost, effectiveness, and cost-effectiveness based on probabilities, costs, and benefits derived from the literature [63]. Using IADPSG guidelines proved more expensive but also more effective and more cost effective ($61,503/quality adjusted life year (QALY)). Sensitivity analysis determined that preeclampsia and CS were the two outcomes that had the greatest impact on cost effectiveness. A decision analytic model showed that IADPSG guidelines were cost effective if the costs to treat GDM were < $2630 and efficacy was at least 74.9% of expected efficacy and if at least 2% more patients are diagnosed with GDM. Similarly, Werner et al. found that the one-step method was also cost effective compared to the two-step method at $20,336 per QALY if postdelivery counseling and behavior modification were provided to reduce future diabetes risks [64].

Duran et al. concluded that €14,358.06 could be saved per 100 women using IADPSG versus CC to diagnose GDM [33••]. They estimated that the new criteria would increase treatment costs by €3753.79, but reduce laboratory costs by €1587.76, and further reduce delivery and NICU costs by €16,336.90. Most cost savings were related to a predicted reduction in CS and NICU admissions.

In a systemic review of these issues, Weile et al. found that 12 out of 100 studies between 2002 and 2013 had full economic evaluations [65]. They emphasized that long-term benefits of treating GDM and preventing maternal DM2 should be included in cost-effectiveness analysis for GDM. However, they were unable to recommend any particular GDM screening and diagnostic criteria for global recommendation, due to the high variability in the methodology and results of the studies.

### Acceptance and Endorsements of IADPSG

The ADA endorsed the IADPSG guidelines in 2011 [66]. Both the Endocrine Society and the WHO adopted them in 2013 [67, 68]. However, ACOG continued to recommend the two-step method in a 2011 Committee Opinion [69] and in their 2013 Practice Bulletin citing the concern that approximately 18% of women would be diagnosed with GDM based on IADPSG guidelines [70]. The ACOG 2013 position was based in part on recommendations from the NIH Consensus Conference.

## Remaining Debates Over the Application of Diagnostic Criteria

### Universal Screening Versus Selective Screening

Both the 50-g GCT and the OGTT are unpleasant, time-consuming, and expensive tests. If selective screening based on risk factors could reliably predict those not at risk for GDM, it would reduce costs and patient discomfort. Risk factors for GDM differ depending on criteria used. ACOG includes previous history of GDM, previous history of macrosomia, maternal age, maternal obesity, ethnicity, family history of DM2, multiple gestation, and polycystic ovarian syndrome [70]. While risk factor screening for GDM can be effective at identifying women at risk, the prevalence of these risk factors is unfortunately so high in the USA that using the ADA risk factor criteria [71] as endorsed by ACOG [70] would result in ≥ 90% of women requiring screening [72].

Another study demonstrated that 91% of women with GDM and 80% of women with NGT had one or more risk factors for GDM [60•]. The prevalence of risk factors in the general population varied depending on which risk factor criteria were being used with ADA having better sensitivity and specificity than NICE and Irish, but still only achieving sensitivity 80% and specificity 44% when BMI ≥ 25 kg/m^2^ was included as a risk factor [73].

In Nigeria, selective screening would have missed 20% of GDM cases [30]. ACOG acknowledged that as only 10% of the population would have been exempted from screening based on risk factors many physicians may elect to screen all pregnant patients as a practical matter [24].

### First Trimester Screening of Preexisting Diabetes and the Early Diagnosis of GDM With Worse Outcomes

Studies using either HbA1c, FPG, or FPG followed by a 75-g 2-h OGTT (IADPSG criteria) support early pregnancy screening to diagnose women with PEDM, at high risk of GDM later in pregnancy, greater need for pharmacotherapy, and increased risk of poor outcomes [74–77].

Early pregnancy A1c ≥ 41 mmol/mol (5.9%) is associated with worse outcomes including increased risk of congenital malformations, preeclampsia, shoulder dystocia, and perinatal death [74]. It identified all the patients in the cohort who had overt diabetes. Women with A1c ≥ 41 mmol/mol (5.9%) represented only 2.9% of the total cohort. However, among women with A1c ≥ 41 mmol/mol (5.9%), early in pregnancy, 74% had GDM (diagnosed by IADPSG/WHO criteria either early or later in pregnancy) or overt diabetes (10.4% of this group). As noted above, the sensitivity of diagnosing women with overt diabetes was 100%. However, the sensitivity was not as good at diagnosing GDM, as 12% of women with A1c < 41 mmol/mol (5.9%) met the criteria for GDM earlier than 20 weeks and 12.8% met criteria later than 20 weeks, underscoring the need for either a one-step or two-step screen at 24–28 weeks in women with an early pregnancy A1c < 41 mmol/mol (5.9%).

While IADPSG recommended that first trimester FPG ≥ 5.1 mmol/l (92 mg/dl) be consistent with the diagnosis of GDM, a study by Zhu and Yang demonstrated that FPG declines in pregnancy, so that a first trimester FPG < 6.1 mmol/l (110 mg/dl) is less predictive of GDM later in pregnancy [75]. While first trimester FPG 5.1–6.1 mmol/l (92 to < 110 mg/dl) is also associated with a higher risk of GDM, the incidence may not be sufficient to warrant early detection of GDM. The authors recommend identifying women with FPG 6.1–6.9 mmol/l (110–125 mg/dl) for early intervention, as 66.2% will develop GDM later in pregnancy. They suggest this as a practical approach as there is no RCT outcome data to support early intervention in this intermediate range of glucose values.

A RCT designed to pick the best approach to screening for first trimester GDM compared FPG values 5.1–6.9 mmol/l (92–125 mg/dl), followed by parallel randomization, to either a two-step 50-g (7.8 mmol/l (140 mg/dl) threshold) followed by a 100-g OGTT (CC thresholds) or a one-step 75-g 2-h OGTT (IADPSG criteria) and then rescreening at 24–28 weeks using the two-step method for initial GDM screen negatives [77]. ROC demonstrated that, in the first trimester, the one-step 75-g OGTT was a better predictor for GDM than the FPG test and two-step OGTT. The FPG test with a lower threshold of 5.1 mmol/l (92 mg/dl) had more false-positive test results and was, therefore, less specific than the two other tests.

A study compared early screening at <24 weeks using either A1c 39–46 mmol/l (A1c 5.7–6.4%) or FPG 5.1–6.9 mmol/l (92–125 mg/dl) followed by a one-step 75-g OGTT with IADPSG criteria at 24–28 weeks (early screen group) to the standard two-step method with CC criteria at 24–28 weeks (standard group) [76]. Prevalence of GDM in the early screening group was 9.4% versus that in the standard group 5.3%. A greater percentage of women required pharmacotherapy in the early screening group.

### The Role of FPG Versus OGTT Versus A1c in Screening for GDM in the Third Trimester

The FPG is a screening test candidate because of its high reproducibility [78].

In the HAPO study, risks of some adverse outcomes were low when FPG was ≤ 4.4 mmol/l (80 mg/dl) [4]. However, it was thought that using FPG to potentially identify pregnancies at a very low risk for GDM and for adverse outcomes required further evaluation.

A systematic review for the United States Preventive Services Task Force (USPSTF) compared the FPG, A1c, and 50-g GCT at 24 weeks, as GDM screening tests. A FPG of 4.7 mmol/l (85 mg/dl) had a sensitivity of 87% and a specificity of 52% [79]. In comparison, the GCT with a threshold of 7.8 mmol/l (140 mg/dl) had sensitivity of 70–88% and specificity of 60–89%. A threshold of 7.2 mmol/l (130 mg/dl) had a sensitivity of 88–99% and specificity 66–77%. A1c had the poorest test characteristics.

A retrospective study from China looked at outcomes of more than 25,000 women with FPG ≤ 4.4 mmol/l (80 mg/dl) with treated versus untreated GDM by IADPSG criteria [80]. There was no difference in macrosomia (6.9 vs 7.2%) or neonatal hypoglycemia treated (2.0 vs 1.7%) in treated versus untreated pregnancies, respectively. There was an increased rate of CS in untreated GDM women (treated 48.4% vs untreated 59.7%).

In a study of more than 1300 women, a strategy used the FPG threshold > 5.0 mmol/l (≥ 92 mg/dl) (15.4% of the population) as the initial screening test to rule in GDM to reduce the need for OGTT and proceeding with full screen if FPG ≤ 5.0 mmol/l and BMI > 25 and age > 30 years [37]. This would reduce full screening to 18.7% of the population achieving sensitivity 72.3% and specificity 79.8%.

The strategy of Trujillo and others used the IADPSG criteria to rule in GDM with FPG ≥ 5.1 mmol/l (92 mg/dl) (15.2% or the population) and rule out GDM with FPG ≤ 4.4 mmol/l (80 mg/dl) (54.3% of the population) [81]. Only 38.7% of those with glucose in between these upper and lower limits for FPG required OGTT screening, eliminating the need in 61.3% of women. Sensitivity for diagnosing GDM by this method was 96.9%. Sensitivity to predict adverse outcomes was 60%.

Agarwal and others found that FPG could be used to diagnose GDM in 82.7% of a predominantly Arabic population [62•]. Similar to the study by Trujillo et al., this study used a FPG followed by a 75-g OGTT, if needed. If FPG < 4.4 (80 mg/dl) rules out GDM or ≥ 5.1 mmol/l (92 mg/dl) rules in GDM, then no additional testing is required. This avoids the need for 50% of the OGTTs. The authors point out that in high-risk populations GCT adds an unnecessary screen and risks missing 20% with a false negative screen as well as those who do not show for the two-step OGTT (18% in this study). Proceeding with a diagnostic test but using the fasting value to rule in or rule out GDM eliminates the need for the OGTT in 50%. It is important to note that this is a predominantly Arabic population and there is variation in the fasting versus postprandial glucose patterns based on ethnicity.

Sevket et al. looked at the utility of the A1c in the diagnosis of GDM at 24–28 weeks’ gestation [82]. GDM was diagnosed by IADPSG criteria, and a rule in/rule out algorithm was used to evaluate the utility of A1c at 24 weeks as a screening tool to reduce the number of OGTTs. A lower A1c threshold of 27 mmol/mol (4.6%) and higher threshold of 39 mmol/mol (5.7%) were determined based on ROC. Sensitivity using these thresholds was 94.4%, but 33% would have been misclassified as having GDM. The algorithm only reduced the need for OGTT by 25%. The authors concluded that the A1c is not a good screening test for GDM.

To summarize, the fasting glucose, as an initial screen for the one-step method to rule out GDM with FPG < 4.4 (80 mg/dl) and to rule in GDM with FPG ≥ 5.1 mmol/l (92 mg/dl), reduces the need for OGTT by 50%. It is a better screening tool than the GCT of the two step method which has 20% false negatives. The A1c is a poor screening test for GDM at 24–28 weeks.

## Conclusions

Currently, ACOG lists both the NDDG and CC criteria for the diagnosis of GDM [70]. The ADA acknowledged the GDM screening and diagnosis controversy in their 2015 Practice Guidelines, and now recommends either the one-step IADPSG or the two-step method (either the NDDG or CC criteria) preferred by ACOG. However, recent data support the use of the CC criteria over NDDG criteria [13]. Outside the USA, the WHO and the International Federation of Gynecology and Obstetrics (FIGO) support the use of the IADPSG criteria [83]. Other varieties of responses to the IADPSG recommendations from the other nations also demonstrate the complexity and controversy, especially given concerns around access to healthcare and costs due to increases in the prevalence of GDM with the one-step IADPSG diagnostic criteria.

Studies of outcomes of women diagnosed with GDM based on the IADPSG criteria excluding other usual criteria demonstrate worse outcomes than those with NGT indicating a likely opportunity to improve outcomes with treatment. RCTs of treatment of GDM demonstrate reductions in adverse pregnancy and neonatal outcomes with treatment, although these studies did not use the exact GDM thresholds proposed by the IADPSG [14, 48]. The financial implications of IADPSG may be offset by lowering costs of diagnosis and treatment and improving effectiveness of treatment, particularly in higher risk populations. Counseling about long-term risks of diabetes and lifestyle prevention should be included in the treatment plan to further improve cost effectiveness. Universal screening has better sensitivity than risk factor screening. Appealing strategies to reduce the cost of screening that makes use of the fasting glucose in a rule out < 4.4 mmol/l (80 mg/dl) or a rule in ≥ 5.1 mmol/l (92 mg/dl) manner and proceeding with full diagnostic testing for in-between values provide high sensitivity and reduce the need for a full diagnostic testing in 50% of women. The question of which 75-g 2-h OGTT glucose thresholds to use will continue to be debated due to the linear relationship between adverse pregnancy outcomes and glycemia. Use of the current IADPSG thresholds set at OR 1.75 of mean outcomes of HAPO with one abnormal value seems reasonable but small upward adjustments toward CC thresholds may help drive consensus.

First trimester screening methods may include the use of an A1c cutoff of 41 mmol/mol (5.9%) to identify all women with preexisting diabetes and women with high risk of GDM and adverse events. A first trimester fasting glucose threshold of ≥ 6.1 mmol/l (110 mg/dl) may also be used to screen women with high risk of future GDM. Lower glucose targets 5.1–6.0 mmol/l (92–109 mg/dl) recommended by IADPSG 2010 may have poor specificity to diagnose future GDM. Women who screen negative in the first trimester should be rescreened at 24–28 weeks. The role of the 50-g GCT for screening women who have already been identified as high risk of GDM in the first trimester is not substantiated. These women should go directly to diagnostic testing. Uses of local, regional, and institutional diagnostic criteria persist but steps should be taken to broaden consensus to use the one-step IADPSG criteria.
